# Ventricular assist device outflow graft in congenitally corrected transposition of great arteries - a surgical challenge

**DOI:** 10.1186/1749-8090-7-93

**Published:** 2012-09-26

**Authors:** Prashant N Mohite, Aron F Popov, Diana Garcia, Rachel Hards, Bartlomeij Zych, Asghar Khaghani, Andre R Simon

**Affiliations:** 1 Department of Cardiothoracic Transplantation & Mechanical Support, Royal Brompton and Harefield NHS Trust, Harefield Hospital, Hill End Road, Harefield, Middlesex UB9 6JH, UK

**Keywords:** Congenitally corrected transposition of great arteries, Transposition of great arteries, Ventricular assist device

## Abstract

Congenitally corrected transposition of the great arteries is a complex congenital cardiac anomaly with a wide spectrum of morphologic features and clinical profiles. Most patients are diagnosed late in their life, undergoes surgical repairs, eventually leading to systemic ventricular failure needing heart transplant or mechanical circulatory assistance. As, aorta is located anterior to and left of the PA (Transposition of great arteries), the outflow graft of ventricular assist device traverse across pulmonary artery to reach aorta which poses challenge during further surgical explorations.

## Background

Congenitally corrected transposition of the great arteries (CCTGA) is a complex congenital cardiac anomaly with a wide spectrum of morphologic features and clinical profiles. Most patients are diagnosed late in their life, undergoes surgical repairs, eventually leading to systemic ventricular failure needing heart transplant or mechanical circulatory assistance. Normally, the outflow graft of the ventricular assist device (VADs), especially with new axial flow devices, ejects from the device and navigates by the right side of the heart to terminate into the ascending aorta. Due to the anatomical anomaly we describe a modificated surgical technique for positioning the ventricular assist device outflow graft.

## Case presentation

A 53 year old gentleman was diagnosed of congenitally corrected transposition of great arteries (CCTGA) with pulmonary stenosis in his thirties. He had DDD pacemaker implanted at that time for atrio-ventricular block. He was normally fit and active but had over the previous six months noticed a reduction in his effort capacity. His heart failure therapy was intensified with beta-blocker, ACE inhibitor and diuretics, despite he gradually deteriorated to NYHA class III. Echocardiography revealed moderately dilated, hypertrophied systemic ventricle with significant systolic impairment (Ejection Fraction of 14%). There was moderate tricuspid regurgitation. There was incoordination of both ventricles. It was decided to implant left ventricular assist device (LVAD) for significant systemic ventricular dysfunction secondary to CCTGA.

Chest was opened by sternotomy and patient was put on cardiopulmonary bypass through an arterial cannula in ascending aorta and two-stage venous cannula in right atrium. Heartmate II teflon ring was attached to the systemic ventricle using 16 pledgeted Polyester (Ethibond Excel) 2–0 sutures. The myocardium within the ring lumen was resected using coring knife and Heartmate II inflow cannula was then inserted through it. The outflow graft was anastomosed to ascending aorta using side biting clamp with a 4–0 running Polypropylene suture and de-aired by releasing clamps before knotting. The LVAD was started as CPB weaned off slowly to generate flows of 4 to 5 l/min. The post-operative course was smooth and the patient was ambulant on 4^th^ day and his long term goal remained heart transplantation.

**Figure 1:**
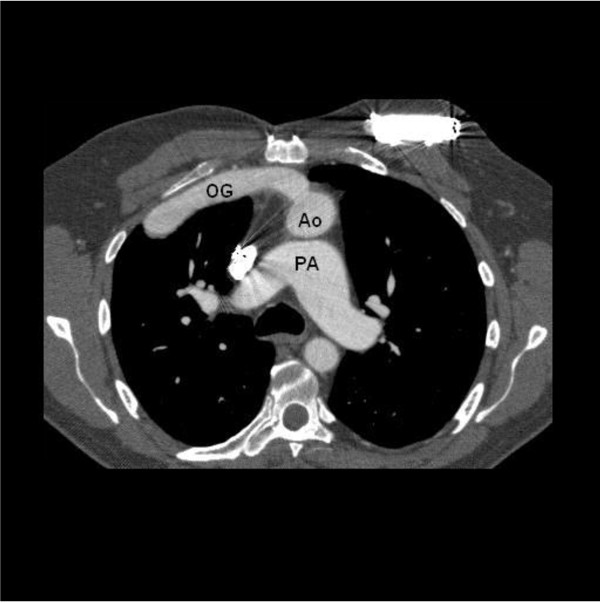
Contrast CT showing position of Outflow graft (OG) in relation to pulmonary artery (PA) and aorta (Ao).

**Figure 2:**
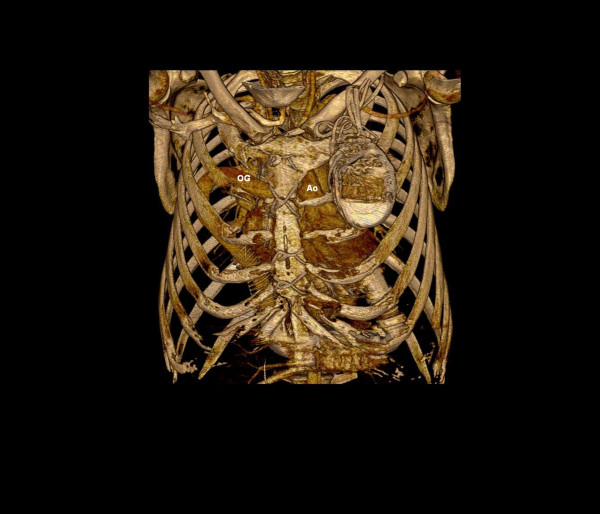
Reconstruction of CT images showing outflow graft (OG) passing behind the sternum before anastomosing aorta (Ao).

A computed tomography (CT) of the thorax was done in routine follow up after 6 months which showed the outflow graft of LVAD passing over Pulmonary artery (PA) under the upper sternum, transversing to the left and anastomosing to the side of aorta (Figure 1). Reconstruction of CT images showed the whole course of the graft (Figure 2). The graft was passing from right to left side under the upper sternum to reach the aorta which is abnormally situated on the left side of the PA.

## Discussion

Adults with congenital heart defects and congestive heart failure are a challenging population because of its complex anatomy, prior surgical palliation, and hemodynamic status. Advances in palliation of congenital heart disease have resulted in improved survival to adulthood. Many of these patients ultimately develop end-stage heart failure requiring heart transplant or LVAD implantation.

The CCTGA is characterized by atrio-ventricular and ventriculo-arterial discordance in which morphologic right ventricle functions as the systemic ventricle (SV), whereas the morphologic left ventricle functions as the pulmonary ventricle (PV). In this anomaly, the systemic atrio-ventricular valve (SAVV) is a morphologic tricuspid valve, whereas the pulmonary atrio-ventricular valve (PAVV) is a mitral valve [1,2]. The great arteries are transposed, with the aorta rising from the RV and the PA rising from the LV. The aorta is located anterior to and left of the PA; thus, the prefix of ‘L’ is used [3]. Despite adequate repair, patients with systemic RVs have an increased risk for developing heart failure accompanied by a high mortality rate [4].

At the time of referral for surgical repair, the majority of these patients have significant SV dysfunction with advanced symptoms. Although excellent early surgical results can be achieved, residual impairment of the SV is common and may eventually necessitate cardiac transplantation [5,6]. Systemic ventricular failure is a known complication after a Senning or Mustard procedure because the morphologic RV must function as the systemic ventricle [7].

Development in the field of mechanical circulatory assist devices in recent decades offer an additional option for patients of end stage heart disease with various aetiologies including congenital heart disease [8]. If rapid deterioration of cardiac function ensues before a donor heart becomes available, the use of an LVAD may be the only option for these patients. Implantation of an LVAD in a patient with TGA after surgical repair was first described by Wiklund et al., who achieved a successful outcome using a HeartMate device [9]. Several cases of VAD implantation using different generations of device are reported in the literature [10,11].

Normally, the outflow graft of the VADs, especially with new axial flow devices, ejects from the device and navigates by the right side of the heart to terminate into the ascending aorta. Both in TGA and CCTGA, the aorta is located anterior to and left of the PA with various degrees of malrotation. Hence, the outflow graft has to traverse across the PA from right to left to reach the ascending aorta. This brings the graft under the sternum in close proximity. Reoperation is needed these cases for VAD explant, VAD up-gradation, pulmonary VAD implantation or eventual heart transplant. Re-entry through the median sternotomy is unavoidable under such circumstances. The outflow graft lying under the sternum yields a great risk of cutting through the graft which may leads to catastrophic haemorrhage and exsanguinations.

Different techniques of reinforcing and positioning the outflow graft to make them less vulnerable at re-do sternotomy like tunnelling the graft in PTFE patch or through the right pleural cavity were reported [12]. However, these techniques protect the graft in presence of normal heart anatomy. In case of TGA due to anomalous relation of great arteries, the outflow graft crosses midline over PA and comes in contact with sternum. The injury at the time of re-do sternotomy can be avoided by approximating the pericardium over the graft, but there remains possibility of graft obstruction due to pericardial tension. The thymic fat over the pericardium can be mobilised and approximated over the graft. Use of Gore-Tex, PTFE or Teflon sheet as a pericardial substitute in cases of major cardiovascular operation to avoid injury during re-do sternotomy is well reported [13]. Its use in LVAD surgery is not yet published, although can be used in cases with graft coming in contact with sternum. Another way to protect the outflow graft is to tunnel it under superior vena cava before anastomosing over ascending aorta [14]. If none of the techniques of graft protection were utilized at the time of LAVD implantation, femoro-femoral cardiopulmonary bypass and a careful dissection through right second or third intercostal space before re-do sternotomy is essential.

## Conclusion

Ventricular assist device implantation in patients with congenitally corrected transposition of great arteries is a technical challenge; however it can be performed in experienced transplantation units with an acceptable postoperative result.

## Consent

A written informed consent was obtained from the patient’s next of kin for publication of this case report and accompanying images. A copy of the written consent is available for review by the Editor-in-Chief of this journal.

## Competing interests

The authors declare that they have no competing interests.

## Authors’ contributions

PM wrote the manuscript, AP co-wrote the manuscript and managed figures; BZ and DG were involved in the concept, RH made critical revision of the manuscript, AK and AS approved the manuscript. All authors read and approved the final manuscript.
